# Effect of the tip state during qPlus noncontact atomic force microscopy of Si(100) at 5 K: Probing the probe

**DOI:** 10.3762/bjnano.3.3

**Published:** 2012-01-09

**Authors:** Adam Sweetman, Sam Jarvis, Rosanna Danza, Philip Moriarty

**Affiliations:** 1 School of Physics and Astronomy, University of Nottingham, Nottingham NG7 2RD, U.K.

**Keywords:** force spectroscopy, image contrast, noncontact AFM, qPlus, Si(001), Si(100), tip (apex) structure

## Abstract

**Background:** Noncontact atomic force microscopy (NC-AFM) now regularly produces atomic-resolution images on a wide range of surfaces, and has demonstrated the capability for atomic manipulation solely using chemical forces. Nonetheless, the role of the tip apex in both imaging and manipulation remains poorly understood and is an active area of research both experimentally and theoretically. Recent work employing specially functionalised tips has provided additional impetus to elucidating the role of the tip apex in the observed contrast.

**Results:** We present an analysis of the influence of the tip apex during imaging of the Si(100) substrate in ultra-high vacuum (UHV) at 5 K using a qPlus sensor for noncontact atomic force microscopy (NC-AFM). Data demonstrating stable imaging with a range of tip apexes, each with a characteristic imaging signature, have been acquired. By imaging at close to zero applied bias we eliminate the influence of tunnel current on the force between tip and surface, and also the tunnel-current-induced excitation of silicon dimers, which is a key issue in scanning probe studies of Si(100).

**Conclusion:** A wide range of novel imaging mechanisms are demonstrated on the Si(100) surface, which can only be explained by variations in the precise structural configuration at the apex of the tip. Such images provide a valuable resource for theoreticians working on the development of realistic tip structures for NC-AFM simulations. Force spectroscopy measurements show that the tip termination critically affects both the short-range force and dissipated energy.

## Introduction

It is now generally accepted that atomic resolution in NC-AFM imaging on semiconducting surfaces is due to the chemical force between the atoms of the surface and the last few atoms of the tip apex [1–4]. Even with well-prepared tips and surfaces, however, a wide range of imaging interactions are often observed, resulting in varying apparent topographic structures [5–7]. In cases where there has been debate as to the surface structure (for example TiO_2_ [8] and Si(100) [9]) different imaging mechanisms can result in inconclusive, or even erroneous results. Consequently, there has been a considerable effort of late to model the tip–surface interaction in NC-AFM by using more-realistic tip structures [10–12], although this requires a considerable computational expense. These efforts are, however, often hampered as there can be a reticence to publish results showing imaging that cannot be easily understood, with a perhaps understandable preference to present data which fits accepted interaction models. In this paper, by highlighting a wide range of observed behaviours, we hope to provide valuable information to the modelling community that will lead to the investigation of more-realistic tip structures, and their respective tip–sample interaction and contrast mechanisms.

The role of the tip was investigated by imaging the well studied Si(100) surface. The Si(100) surface is now understood to form a stable c(4 × 2)/p(2 × 2) reconstruction at temperatures below 120 K [9,13–15], but enjoyed lively debate in the literature for some time due to conflicting results [9]. Si(100), while structurally well understood for some time, is unusually sensitive to the influence of temperature and, importantly, the probe [14] (especially the influence of tunnelling electrons during STM). While in principle NC-AFM can provide a “cleaner” system (i.e., imaging is possible without the presence of tunnelling electrons), a bias is often applied to null out the contact-potential difference (CPD) between tip and sample. This may, however, also perturb the system through the influence of the inelastic scattering of tunnelling electrons. In addition, it has recently been shown that the presence of significant tunnel currents can influence the tip–sample force during NC-AFM imaging of semiconductor surfaces [16–17], and hence significantly complicate the interpretation of the acquired images.

**Figure 1:**
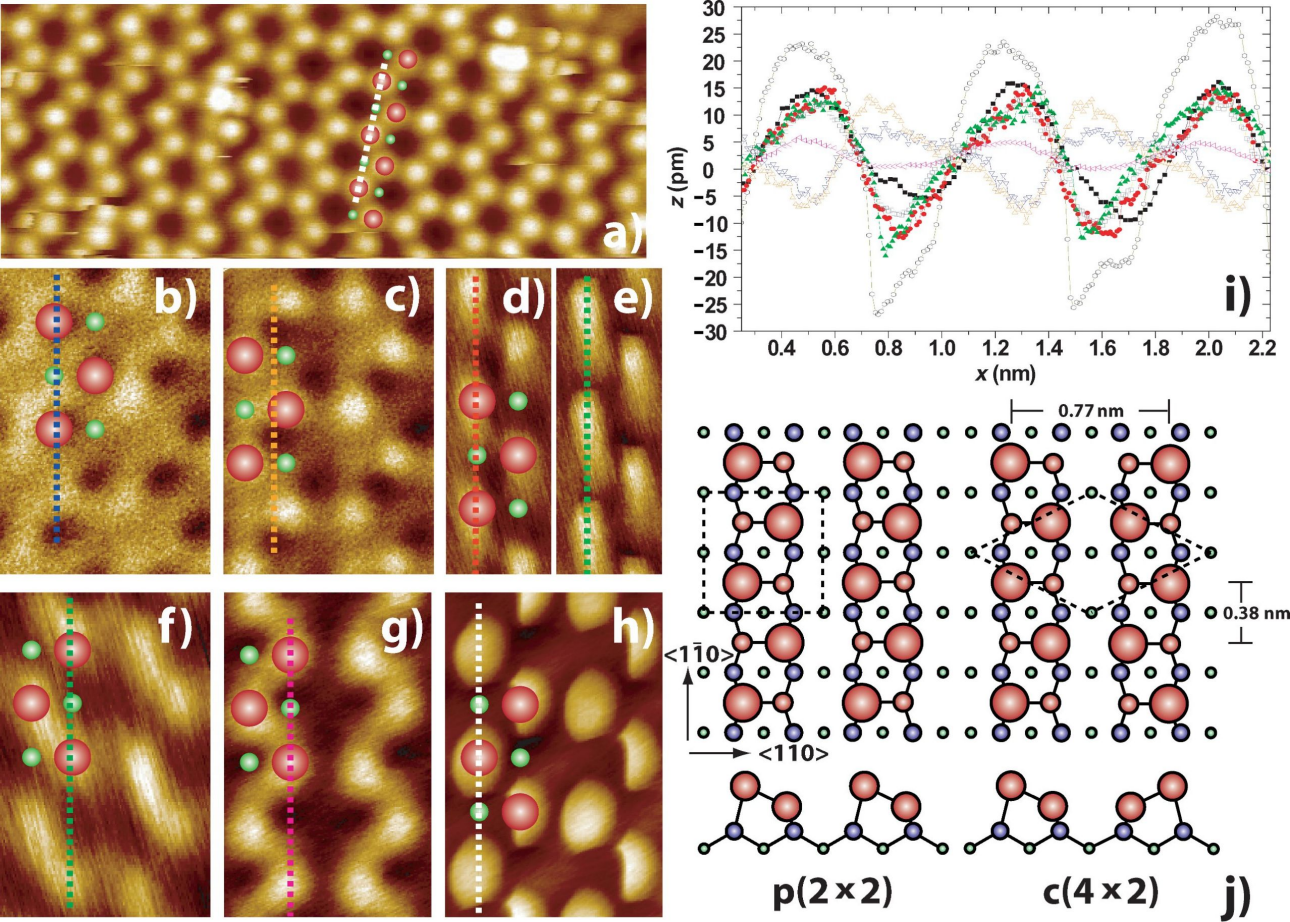
Topographs acquired in constant Δ*f* NC-AFM of Si(100) at 5 K, demonstrating different imaging mechanisms. Images have been rotated to align the direction of dimer rows. (a) High quality “conventional” image, “slicing” of some atoms indicates scan-induced dimer manipulation [19] Image size 8.9 nm × 3.5 nm. Δ*f_set_* = −46 Hz, *A*_0_ = 0.25 nm. (b) “Inverted” image. Δ*f_set_* = −53 Hz, *A*_0_ = 0.1 nm. (c) Subsequent scan of the same region as (b) with the same tip apex at higher Δ*f* setpoint showing a “depression/protrusion” double image. Δ*f_set_* = −54 Hz, *A*_0_ = 0.1nm. (d) and (e) “Dimer”-type image showing the difference between the forward (d) and reverse (e) scan directions. Δ*f_set_* = −10 Hz, *A*_0_ = 0.25 nm. (f) “Crescent”-type image. Δ*f_set_* = −40.5 Hz, *A*_0_ = 0.25 nm. (g) “Wormlike” image. Δ*f_set_* = −10.4 Hz, *A*_0_ = 0.25 nm. (h) “Discuslike” image. Δ*f_set_* = −44 Hz, *A*_0_ = 0.25 nm. In each image the dotted line shows the location of the line profiles and the illustration shows the apparent position of the atoms in the c(4 × 2) reconstruction (large red - “up” atoms, small green - “down” atoms). Image size (b), (c) and (f)–(h) 1.4 nm × 2.1 nm, (d) and (e) 0.8 nm × 2.1 nm. (i) Line profiles from positions indicated in (a)–(h). (a) “Conventional” (black filled squares), (b) “Inverted” (empty blue triangles), (c) “Inverted high setpoint” (empty orange triangles), (d) “Dimer” forward (filled red circles), (e) “Dimer” back (filled green triangles), (f) “Crescent” (empty green squares), (g) “Wormlike” (empty pink triangles), (h) “Discuslike” (empty black circles), (j) Ball and stick model of the Si(100) surface reconstruction showing in-phase (p(2 × 2)) and out-of-phase (c(4 × 2)) dimer buckling.

The silicon atoms terminating the Si(100) surface pair up into dimers in order to reduce the number of dangling bonds, and subsequently buckle, forming rows of alternately buckled dimers along the surface (Figure 1j). It has been shown that the structure of the rows may be locally manipulated by controlled tunnel-current injection [18], and we recently demonstrated that the buckling of the dimers can be toggled with atomic precision by direct application of mechanical force during NC-AFM [19–20]. In this paper we present imaging and force spectroscopy of the Si(100) surface at 5 K by qPlus [21] NC-AFM at zero applied bias, and investigate the influence of different apex types on the qualitative image appearance, and quantitative short-range tip–sample force and dissipation.

### Experimental details

We used a commercial low-temperature (LT) STM/qPlus NC-AFM instrument (Omicron Nanotechnology GmbH) operating in UHV (base pressure ≤ 5 × 10^−11^ mbar) cooled to 5 K. The sample and tip-preparation procedures are described in detail elsewhere [19,22]. Briefly, boron-doped silicon samples were prepared by standard flash-annealing to 1200 °C, and then slow cooling from 900 °C to room temperature before being placed into the scan head. We introduced commercial qPlus sensors (Omicron GmbH), with an electrochemically etched tungsten wire attached to one tine of the tuning fork, into the scan head without any ex situ tip treatment. The tips were prepared by standard STM methods (voltage pulses, controlled contacts with the sample) until good atomic resolution was obtained in STM feedback, at which point we made the transition to NC-AFM (i.e., Δ*f*) feedback. As a result of our tip preparation procedures our tips are likely to be silicon- rather than tungsten-terminated, and this assumption is supported by a combined scanning electron microscopy (SEM)/energy dispersive X-ray spectroscopy (EDX) study on an STM tip prepared by similar methods. We imaged at constant Δ*f*, maintaining a constant oscillation amplitude (*A*_0_). All data presented were taken at close to zero bias (i.e., ~0 V applied to the tip, sample held at system ground), in order to eliminate the possibility of electronic crosstalk (Supporting Information File 1) and the effect of tunnelling electrons [19]. To ensure that this was the case, the tunnel current was recorded in parallel for all the results presented below. We detected no DC tunnel current within the noise level of our preamplifier for all images and spectroscopy presented in this paper, nor were we able to detect any AC displacement current (Supporting Information File 1).

## Results and Discussion

### Tip-induced imaging variation

At 5 K we routinely observe the c(4 × 2) reconstruction and associated surface defects (Figure 1a). Note that in order to avoid perturbation of the surface during scanning we typically image at a setpoint corresponding to low tip–sample interaction (i.e., at a frequency shift setpoint just after the onset of atomic resolution) [15,19]. In this “conventional” image the bright circular spots correspond to the “up” atoms of the buckled dimers.

It must be noted that here the word “conventional” is used in the sense of the contrast most commonly reported in the literature, and which most intuitively corresponds to the known topography of the surface, which is not that which is necessarily most commonly observed during experiments. In fact only a small proportion of the contrasts we observe are of this form. Statistical analysis of the relative prevalence of different contrast types is difficult as typically we attempt to coerce the tip state into producing “conventional” images before performing manipulation experiments, so as to simplify interpretation of our experiments. Therefore, simply counting the number of images of each type acquired over an experimental run (in which the purpose of the experiment is not simply to investigate the influence of the tip state) does not provide a good statistical measure as there is an inbuilt bias in the dataset. Nonetheless, an informal measure suggests that upon initial atomic resolution of the surface in NC-AFM (i.e., the first scans with a given apex producing atomic resolution), the probability of obtaining “conventional” resolution is on order of ~50%. Analysing the statistics from different forms of atomic resolution images is a topic under investigation by means of computer-aided tip preparation [23–24].

As stated, we also see a considerable number of additional characteristic image types, which are presented here to highlight the key role of the tip apex in contrast formation, even on well-defined surfaces. First, we consider the case of “inverted” images (Figure 1b). Although we refer to this contrast as “inverted” we also note that similar imaging can occur in the case of “enhanced-depression” images (where the height of the up atoms is reduced, and the dips associated with the down atoms are enhanced [25]). However, it appears that in this case the “up” atoms appear as dark depressions, as we have observed depressions corresponding to known defect-based protrusions on the surface with tips displaying similar inverted contrast over the clean surface (Supporting Information File 1). Inverted images have previously been reported during NC-AFM imaging of Si(111) [16], but in this instance this was likely due to the influence of significant tunnel currents [17], and also during the imaging of adsorbed molecules [4,26–27]. Here, however, an additional subtlety is revealed upon imaging the same region at slightly higher Δ*f* setpoints (Figure 1c). It is now clear that, in addition to the depressions, a corresponding second set of protrusions is evident at a spacing of 0.56 ± 0.01 nm. This suggests an intriguing form of “double tip”, where it appears one apex of the tip has an attractive interaction with the surface atoms, whilst the other (at the same tip–sample separation) is more repulsive.

**Figure 2:**
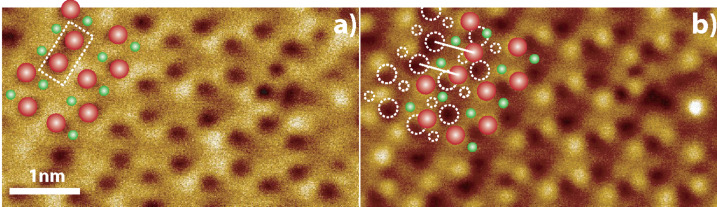
Larger scans of (a) inverted and (b) high-setpoint inverted images presented in Figure 1b and Figure 1c. In a) the large red (small green) circles indicate the apparent location of the up (down) atoms. In (b) the dotted outlines indicate the position of the atoms in the inverted image and the red (green) circles indicate the new apparent position of the up (down) atoms. Solid white lines link the inverted and noninverted images of the same atoms.

The identification of the depressions as “up” atoms was performed by identifying characteristic structures and cross-comparing between the inverted image and the subsequent high setpoint image. This is demonstrated in Figure 2 in which we use a phason buckling defect (2 dimers in a row in the same buckling configuration). By using the defects to correlate the features between the two images it can be seen that in the high setpoint image (Figure 2b) the apex producing the “inverted” image and the apex producing the “conventional” image are offset by 0.56 ± 0.01 nm (c.f., the spacing of the dimer rows 0.77 ± 0.01 nm), which is suggestive of two atoms terminating the tip and exhibiting radically different interactions with the surface, either due to different elemental composition, or a structurally distorted charge density. It should be noted that thermal drift during these scans was negligible (much less than one atomic diameter per scan), and therefore drift is not an issue in the assignment of atomic position. This assignment is confirmed by analysis of other images with similar contrast in which the presence of dopant-related defects [22,28] allows unambiguous identification of true contrast inversion (Supporting Information File 1), we also stress that in the absence of tunnelling electrons this imaging must have a different origin to the image inversion that is due to a tunnel-current-induced force as recently reported by Weymouth et al. [17].

Another imaging type we commonly observe is the so-called “dimer-tip” type image [5–6]. Typically this is characterised by surface atoms appearing elongated and rectangular, and a degree of asymmetry in the imaging (c.f., Figure 1d and Figure 1e), and has been hypothesised to result from a silicon dimer-like termination of the tip apex.

In addition to these previously reported image types, we also observed at least three additional characteristic image types. Figure 1f shows pairs of “up” atoms that appear to be joined to form a “crescent” shaped curved protrusion. This is similar, yet distinct, from Figure 1g, which we term the “wormlike” topography. Here the “up” atoms are clearly resolved but joined together to form a long continuous undulating band along the row. The final image type is shown in Figure 1h. Here the “up” atoms are imaged as flattened discs. In addition we note intriguing, and well-defined, straight edges on some discs, suggestive of a complex polygonal tip apex.

This wide variety of tip apices may, in part, be due to our low temperature operation, which may allow a population of metastable tip structures to exist [29] that might only have short lifetimes at room temperature. Additionally, our STM-based tip preparation may allow us to access a wider apex parameter space than is available from conventionally prepared silicon cantilever AFM tips. Moreover, it must be noted that although we strongly suspect our tips are bulk silicon terminated, at least four atomic species (W and O from the tip and Si and B from the surface) are, in principle, available to terminate the apex, each of which may result in a radically different tip–sample interaction [30].

In each image of Figure 1 the apparent positions of the “up” and “down” atoms of the dimers were assigned by checking the registry of the dimer rows against recognised surface features (such as defects). Line profiles were taken along the dimer row in each case, directly over the apparent positions of the atoms, with the exception of Figure 1c, where the line profile was taken in the same absolute position as Figure 1b to highlight the offset position of the normal and inverted atomic positions.

Analysis of line profiles taken along a dimer row in each topograph provides insight into the differences in tip–sample interaction (Figure 1i). Despite their asymmetry it is clear that both the “dimer” and “crescent” images show a similar corrugation to the “conventional” image. The “wormlike” image, despite looking superficially similar, actually shows a marked reduction in apparent corrugation. In contrast, the “discus-like” image shows a dramatically enhanced corrugation, suggesting (in light of significant dissipation observed during imaging; see Supporting Information File 1) that the tip may be deformed significantly during scanning. The “inverted” image shows a dip in apparent height over the atomic positions, but at higher setpoint the edge of the offset protrusion is evident, with a corrugation similar to that seen in the conventional image. It should be noted that variation in setpoint can also result in variation in measured corrugation. In each case, however, we imaged using a Δ*f* setpoint just below that required to perturb the surface [19]. Consequently, we do not expect the imaging forces between different tips to be significantly different in each case, an assumption confirmed by force spectroscopy experiments (see discussion below). It is instructive to note that direct comparison of the frequency shift setpoints for each image is not a good measure of the site specific (short-range) tip–sample interaction, as the magnitude of Δ*f* is highly dependent on the *macroscopic* radius of curvature of the tip (and indeed any CPD between tip and sample), as well as on the oscillation amplitude. The long-range forces can change dramatically after tip preparation by voltage pulsing or making contact with the surface. We find that regardless of the long range behaviour the maximum short range force (with the exception of “inverted” imaging) between tip and sample is usually in the range of 1–2 nN, with forces at the imaging position of ~0.1–0.5 nN. We find the onset of scan-induced dimer flipping provides a natural comparative measure of the (approximate) short-range interaction forces between different scans, as this occurs at a reasonably well-defined tip–sample interaction force.

### Force spectroscopy

**Figure 3:**
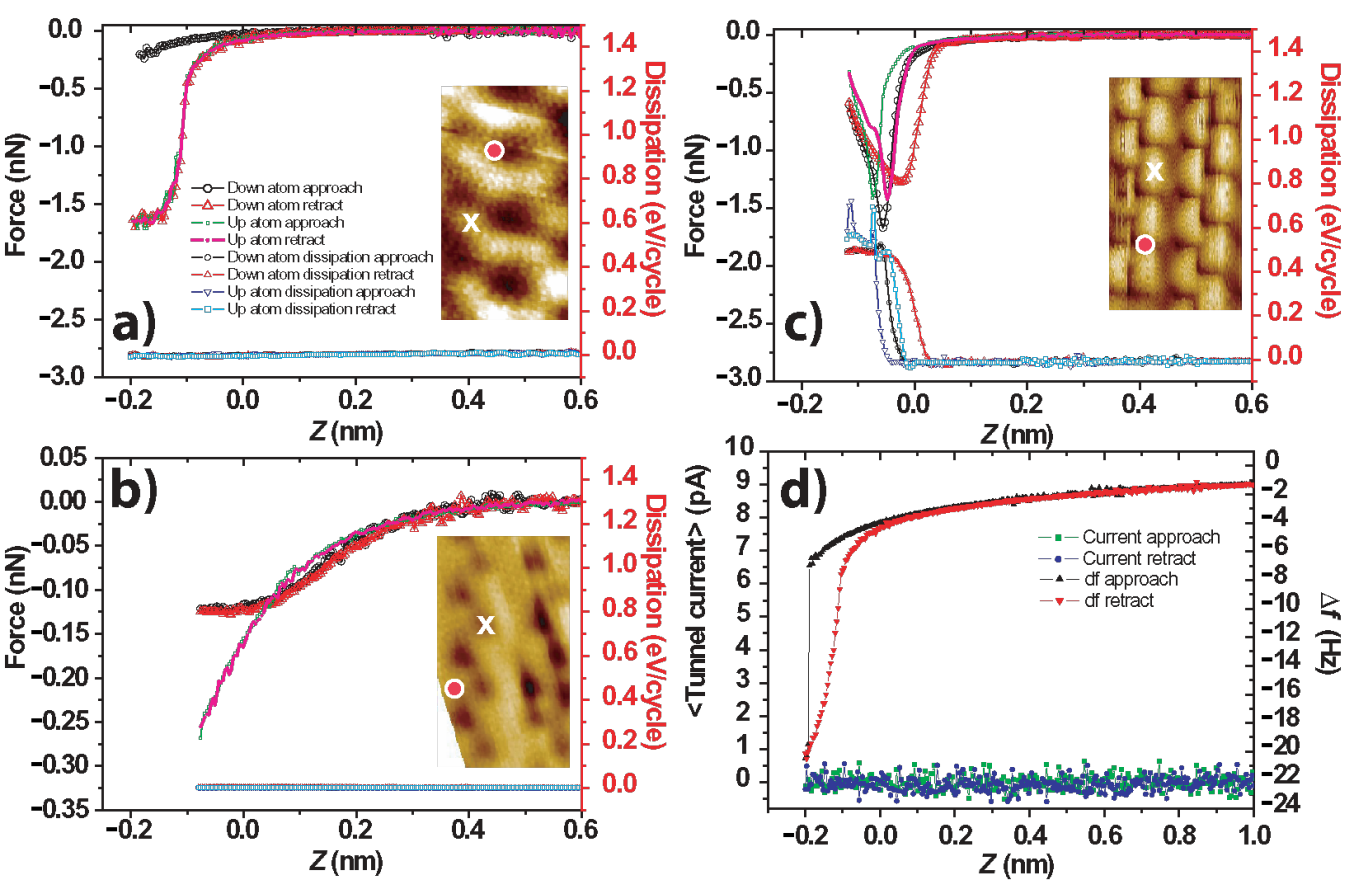
Experimental short-range force (nN) and dissipation (eV/cycle) as a function of relative tip–sample displacement for three different tip apices. The zero in the *z* scale indicates the feedback position. In each case spectroscopy was performed over the apparent location of both an “up’ and “down” dimer atom. Results were obtained with the same probe in one experimental session. (a) Data acquired with a tip demonstrating “conventional” atomic resolution. Δ*f_set_* = −4.3 Hz, *A*_0_ = 0.25 nm (3 × 3 median smoothing applied to inset figure). (b) Data acquired with a tip demonstrating “inverted” atomic resolution. Δ*f_set_* = −5.1 Hz, *A*_0_ = 0.1 nm. (c) Data acquired with a tip demonstrating “dimer” atomic resolution. Δ*f_set_* = −8.4 Hz, *A*_0_ = 0.25 nm. (d) Raw Δ*f* and *I_t_* data corresponding to the spectra in (a). We note that the tunnel current remains zero throughout, as was the case for the spectra in (b) and (c) (data not shown). Note that for (a) and (b) the dissipation signals for each of the spectra overlap within the noise of the data. Insets: Typical imaging for each tip type. Red dot: Location of spectra over “down” atom, White cross: Location of spectra over “up” atom. Keys are the same for each graph.

In order to further elucidate the differences in interaction between different apices, we performed experiments to measure the frequency shift versus z (i.e., Δ*f*(z)) with a number of tip apices. The long-range van der Waals and electrostatic components were removed by fitting the long-range Δ*f* curve to a power law of the form *a*/(*b* + *z*)*^c^* (a detailed discussion of the fitting is found elsewhere [5,19,31]). We inverted the resultant short-range Δ*f* data by using the Sader–Jarvis algorithm [32] to determine the short-range force between the surface and tip apex. In particular we note that the removal of the long-range force by this method may result in significant errors, depending on the quality of the data, range of extrapolation, and determination of short-range cut-off point, amongst other factors. We intend to address in detail the uncertainties associated with short-range force extraction in an upcoming publication [33]. Here, however, we restrict ourselves to the estimation of uncertainties of up to ~30% in the final inverted forces, the main source of this error being the extrapolation required from the long-range power-law fit used to remove the long-range forces. This hinders rigorous quantitative comparison to simulations with different tip apices as the difference in force profiles between simulated force spectroscopy experiments (with different tip apices) are of the same order as the uncertainties associated with the technique for the long-range fitting. Nonetheless, we are able to state that the force profiles associated with the “inverted” image differ significantly from the “dimer” and “conventional” images, and that the observed dissipation varies strongly between different tip apices. The results of these experiments are presented in Figure 3. All of the data presented in Figure 3 were taken during the same experimental session with the same qPlus sensor, therefore only changes in the tip apex would appear to explain the changes in the observed contrast.

In Figure 3a we show typical force spectroscopy data obtained with a tip demonstrating a “conventional” image. Spectroscopy was performed over both “up” and “down” atoms of the dimers. In the case of the “down” atom, spectroscopy produced a correlated dimer-flip event as detailed previously [19]. This results in a significant hysteresis between approach and retract as the atom under the tip changes state. However, over the “up” atom the forward and retract curves overlap within the error of the measurement, indicating that no significant inelastic structural changes occurred on tip or surface. This assumption is supported by the negligible dissipation in both cases [7,34], and we note that the measured forces and behaviour are qualitatively similar to previous results on the same surface [19].

The inverted imaging demonstrates radically different behaviour (Figure 3b). Although the onset of the short-range force occurs over approximately the same range, the turn-around point (i.e., the minimum in the force–distance plot) is almost an order of magnitude smaller than that with the tip producing the “conventional” image. Similar to other reports of inverted contrast we also observe a crossover in the force curves taken over inverted and noninverted regions. In the third set of data, we performed the same experiment with a tip demonstrating “dimer”-type imaging (Figure 3c). Similar to the work of Oyabu et al. on Ge(111) [7], we observe significant dissipation, both at “up”, and “down”, atom positions. Also we note there is significant hysteresis in the force curves, even over the structurally stable “up” atoms, indicating that significant deformations occur at the tip apex during close approach. Interestingly, despite the dramatic differences between the three imaging types, analysis of the force curves reveals that the “imaging force” (i.e., the short-range force between tip and sample at the feedback position) was approximately 0.1–0.2 nN regardless of the image type. We stress that as these results are all obtained in the same session, and in the absence of any tunnel current (Figure 3d), only changes at the tip apex can be responsible for the altered tip–sample interaction.

## Conclusion

In conclusion we have presented data demonstrating a wide range of stable image types in the small-amplitude NC-AFM of the Si(100) surface at 5 K. We have shown that the qualitative and quantitative behaviour of the tip–sample interaction can vary strongly with the same sensor, suggesting that the very apex of the tip dominates the short-range force, and hence the imaging. While we note that the elucidation of the tip structures that produce these contrasts is likely to be nontrivial, we hope that these results will inspire further debate in the modelling community, and help further understanding of contrast mechanisms in NC-AFM imaging of semiconductor surfaces. In particular we note that by operating at zero bias, the influence of tunnelling electrons is eliminated, highlighting the fact that the different types of contrast arise from variations in the short-range covalent interaction between tip and surface. Future experiments related to the controlled functionalisation of the tip may allow us to selectively “tune” the interaction based on the identification of imaging and force-profile signatures, which will be critical for future chemically selective manipulation strategies.

## Supporting Information

Supporting information is available highlighting that no tunnel current was measured during the experiments performed in this paper. We also discuss the assignment of atomic positions due to the double tip present in the high-setpoint “inverted” image presented in Figure 1.

File 1 Complete additional experimental detail and figures

File 2 Representative tunnel current data during zero bias imaging

File 3 Atomic position assignment
