# Supplementary material for: Effect of the tip state during qPlus noncontact atomic force microscopy of Si(100) at 5 K: Probing the probe
Source: Beilstein J Nanotechnol. 2012 Jan 9;3:25–32. doi: 10.3762/bjnano.3.3 (PMC3304327; doi:10.3762/bjnano.3.3)
Supplement: File 1 — Complete additional experimental detail and figures [file Beilstein_J_Nanotechnol-03-25-s001.pdf]

Supporting Information

for

Effect of the tip state during qPlus noncontact atomic force microscopy of Si(100) at 5 K: Probing the probe

Adam Sweetman*, Sam Jarvis, Rosanna Danza and Philip Moriarty

Address: School of Physics and Astronomy, University of Nottingham, Nottingham NG7 2RD, U.K.

Email: Adam Sweetman - adam.sweetman@nottingham.ac.uk; Philip Moriarty - philip.moriarty@nottingham.ac.uk

* Corresponding author

Complete additional experimental detail and figures

Crosstalk elimination

Here we demonstrate that the observed contrast is not a result of electronic crosstalk from the tunnel-current channel and results from a purely physical interaction between the tip apex and surface. In Figure S1 we show the topography, tunnel current, Δf (error signal), and raw damping signals collected for each image from the main paper. In each case there is no contrast in the tunnel-current image. Since there may be a small DC offset in the applied bias (which may vary at the noise level of the DAC interface in the control electronics) it is unlikely that the bias on the tip is exactly 0 V (e.g., as if tip and sample were both directly grounded). Therefore we assign the lack of tunnel current in most of our images to a tip that is effectively insulating at very low biases (perhaps due to having picked up a large quantity of silicon from the surface). However, we do sometimes observe tunnel current signals at very low bias, but this does not appear to perturb the measured Δf or damping signals when the applied bias and measured tunnel current are both small. In any case, for all data in this paper no tunnel

current was detected – in Figure S2 line profiles from two scans are presented showing that there is significant variation in the topography but no detected tunnel current in either instance. We also checked for AC displacement currents induced by the CPD between tip and sample (although with a different tip apex to those used to acquire the results presented in the paper). The displacement current was measured using a lock-in amplifier whilst the tip was oscillating (using a method similar to that described in A. D. Müller et al. Appl. Phys. Lett. 74, 2963 (1999)) at a range of biases (including 0 V). We were unable to detect any displacement current within the noise level of the low-gain preamplifier (~1 pA). Consequently our data (both imaging and force spectroscopy) cannot be assigned to any form of electronic crosstalk and represent the characteristic imaging related to different physical tip apices, and their interaction with the surface.

Calibration of dissipation

The dissipation recorded was determined by measurement of the DC gain of the amplitude regulation (i.e., the damping signal) as described in the Habilitationsschrift of Dr. Franz Giessibl. (Universität Augsburg 2000). The ratio of the free damping to the damping at closest approach is the same as the ratio of the free driving signal to the driving signal at closest approach, and may therefore be used to calculate the dissipated energy by

$$\Delta E_{ts} = 2\pi \frac{E}{Q} \left(\frac{|A'_{drive}|}{|A_{drive}|} - 1 \right) \tag{1}$$

Atom assignment in inverted imaging

Because the Si(100) surface is composed of atoms in two distinct configurations at different absolute heights, particular care must be taken in the interpretation of “inverted” images as depressions may also correspond to the location of the “down”’ atoms . In Figure S3 we show that true contrast inversion is observed by considering the imaging of B-Si dopant-related defect structure [1,2], These ad-dimer structures protrude above the native dimer rows and thus provide a structure that is unambiguously topographically higher than the surrounding atoms. In addition we observe split-off dimers at a step edge, which remain symmetric (i.e., unbuckled) and are thus at (approximately) the same height as “up” atoms of normal buckled dimers. Comparison of conventional high-bias STM, “conventional” imaging, and “inverted” imaging of the same regions (Figure S3) highlights that the protrusions in STM and conventional imaging correspond to the depressions in the inverted image. Although there is weak evidence for a “conventional” double tip over the B-Si ad-dimer during inverted

imaging (as noted in the main paper) we stress that the registry of these features does not match with the position of the noninverted features between rows, and thus the contrast over the clean surface cannot be assigned to the imaging of the down-atom positions. It is possible that the tip structure that produces the inverted contrast may always produce an offset “conventional” image at close approach, and this may in turn provide a valuable clue as the apex structure that produces this contrast. We also note that, in contrast to the data presented in the main paper, a very small tunnel-current signal was detected in some regions of the surface for these images. These are presented alongside the raw topography to highlight that the two signals are well decoupled, and that the variations in imaging are not due to the detected tunnel current.

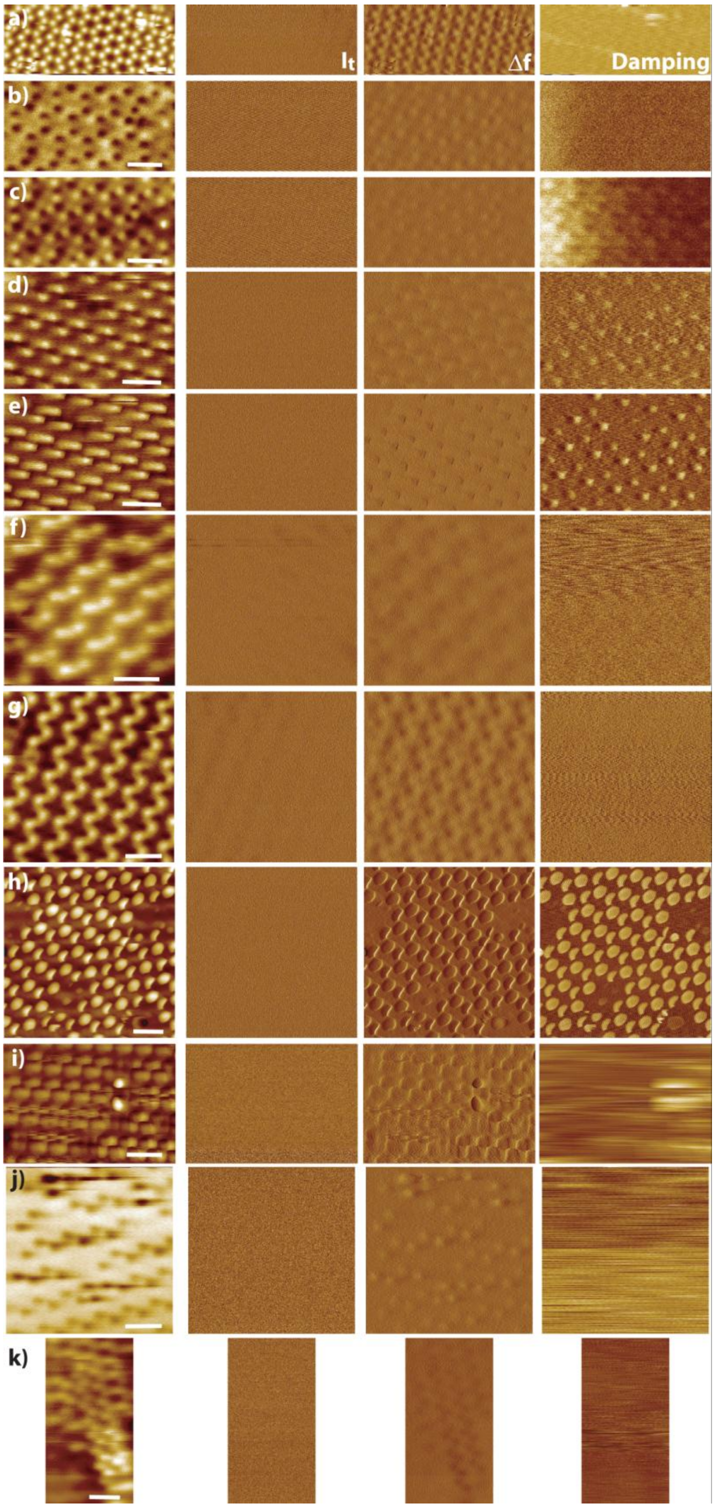

Figure 1: From left to right: complete scans in their original orientations showing topography, tunnel current, Δf (error signal), and raw damping signal for each image presented in the main paper. The scale bar indicates 1nm in all instances. All images have the fast-scan direction left to right and slow-scan direction top to bottom with the exception of row (c) (slow-scan direction bottom to top) and row (e) (fast-scan direction right to left).

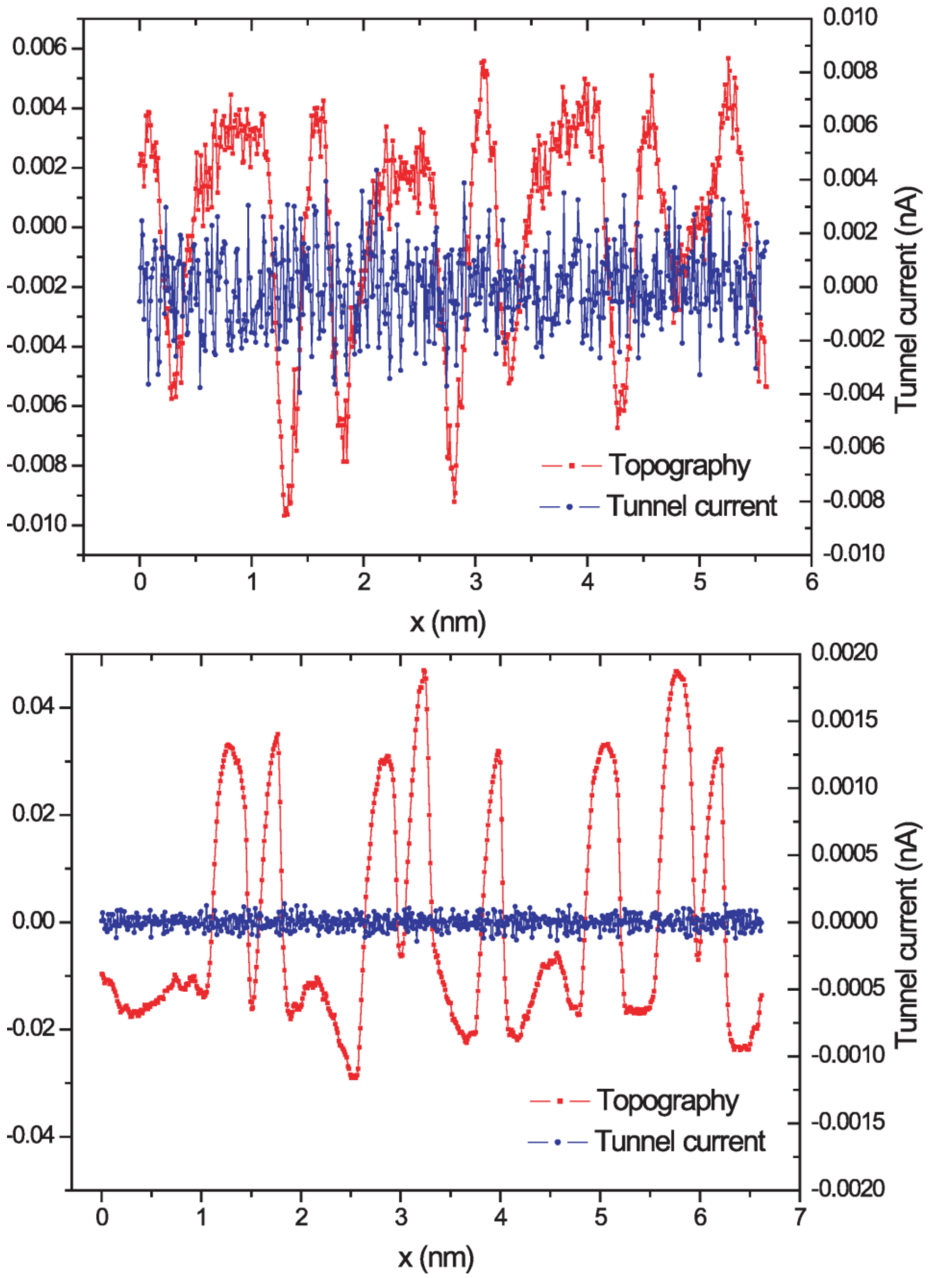

Figure 2: Example line profiles taken perpendicular to the dimer-row direction showing topography and tunnel current from (b), and (h) of Figure S1 respectively.

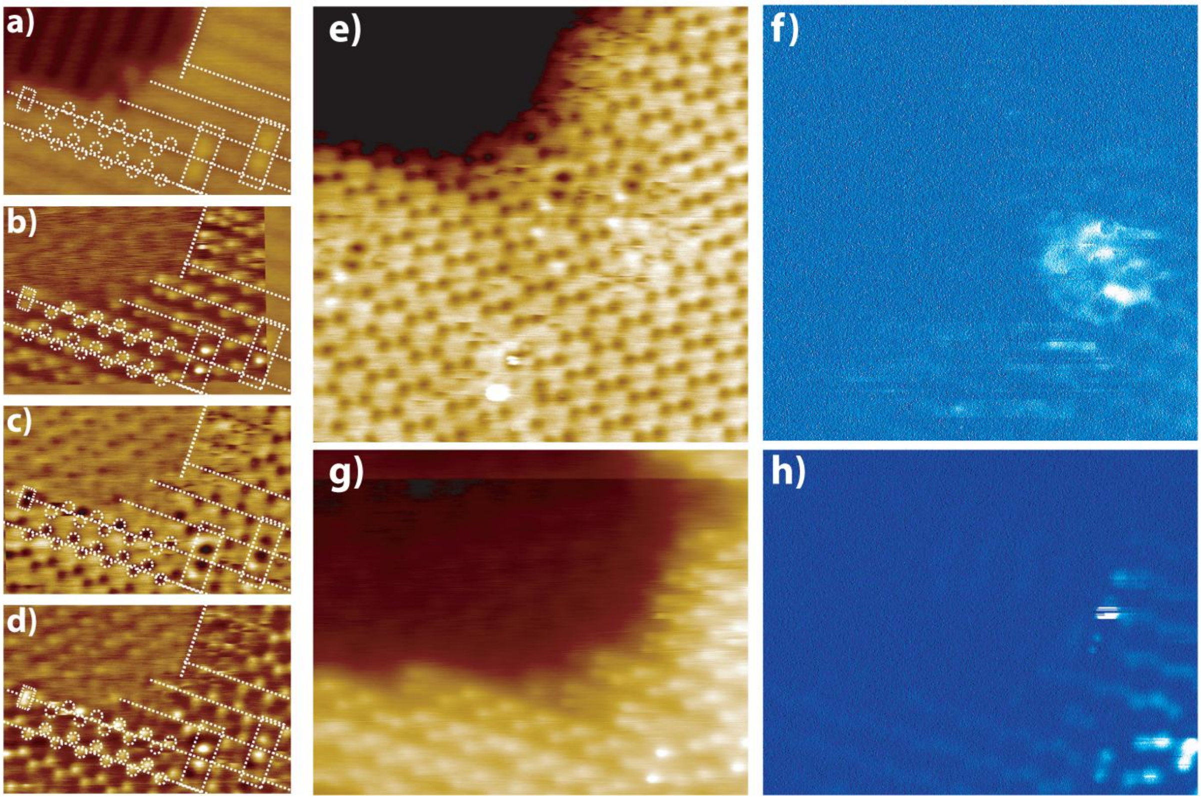

Figure 3: (a) Conventional STM of Si(100) (+2 V, 100 pA) showing B-Si ad-dimer defects (large dotted boxes), a split-off dimer (small dotted box) and step edge (denoted by dotted line). The position of the dimer rows is noted by the dotted lines perpendicular to the step edge. Note at this bias the rows of the surface appear dark, with the bright lines corresponding to the regions in between the dimer rows. (b) Overlay of conventional NC-AFM image (high-pass filtered) of the same region; note the aforementioned defects appear as protrusions. c(4 × 2) periodicity is shown with dotted circles in the position of "up" atoms. Note the presence of buckling defects and scan-induced dimer flipping means the rows do not exhibit perfect c(4 × 2) structure. (c) Overlay of "inverted" NC-AFM image (high-pass filtered) of the same region, note the aforementioned defects now appear as depressions. (d) Contrast-inverted version of (c). (e,f) and (g,h) raw (planesubtracted) topography and tunnel-current images of conventional and inverted contrast, respectively. Levels have been adjusted to maximise contrast on the upper terrace. Range of tunnel current in displayed images ~10 pA, applied bias ~1 mV

References

1. Liu, Z.; Zhang, Z.; Zhu, X. *Phys. Rev. B* **2008,** *77,* 035322. doi:10.1103/PhysRevB.77.035322.
2. Sweetman, A.; Gangopadhyay, S.; Danza, R.; Berdunov, N.; Moriarty, P. *Appl. Phys. Lett.* **2009,** *95,* 063112. doi:10.1063/1.3197595.
