# Supplementary figures and images for: Effect of the tip state during qPlus noncontact atomic force microscopy of Si(100) at 5 K: Probing the probe
Source: Beilstein J Nanotechnol. 2012 Jan 9;3:25–32. doi: 10.3762/bjnano.3.3 (PMC3304327; doi:10.3762/bjnano.3.3)

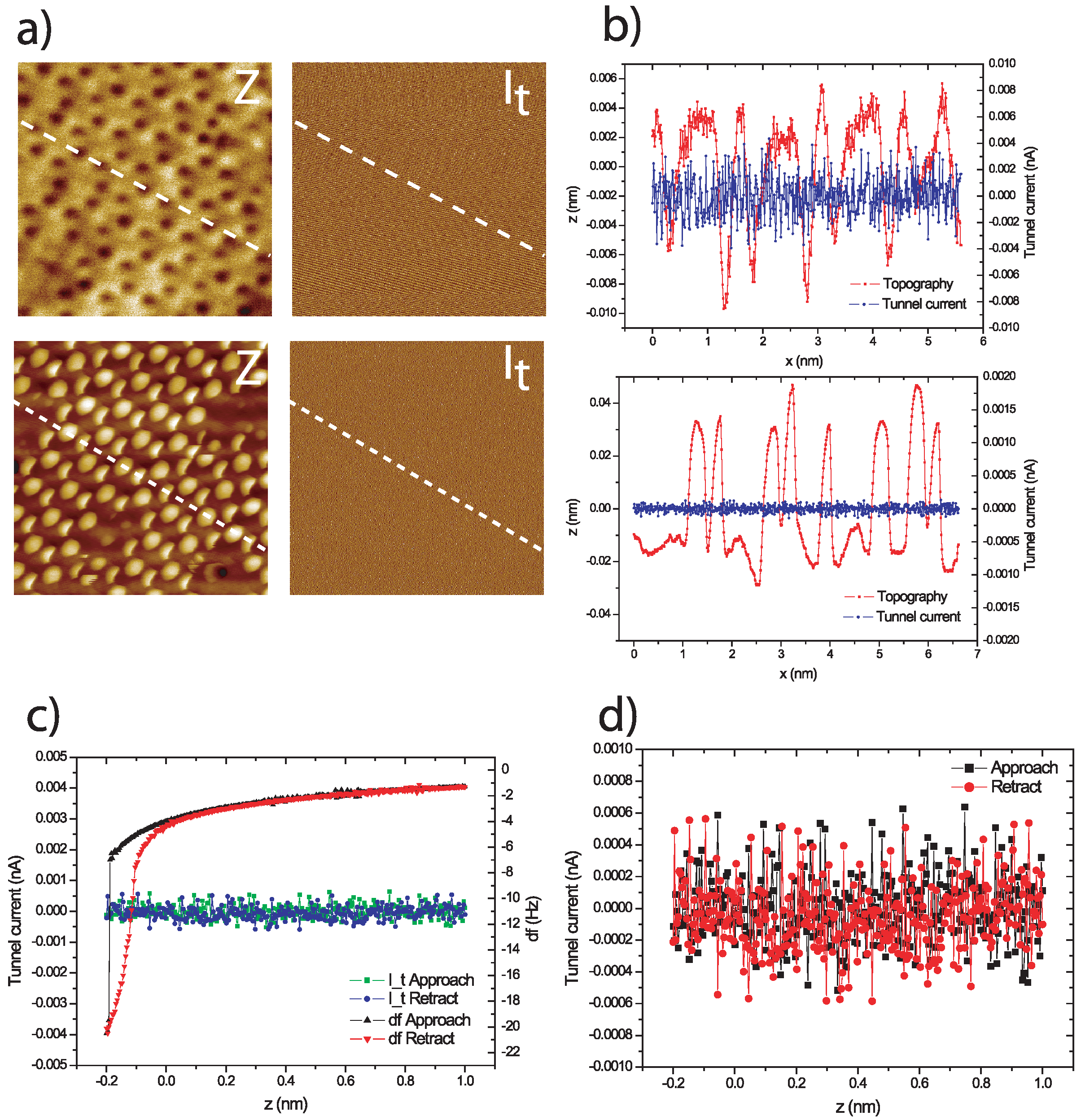

Supplement: File 2 — Representative tunnel current data during zero bias imaging [file Beilstein_J_Nanotechnol-03-25-s002.png]

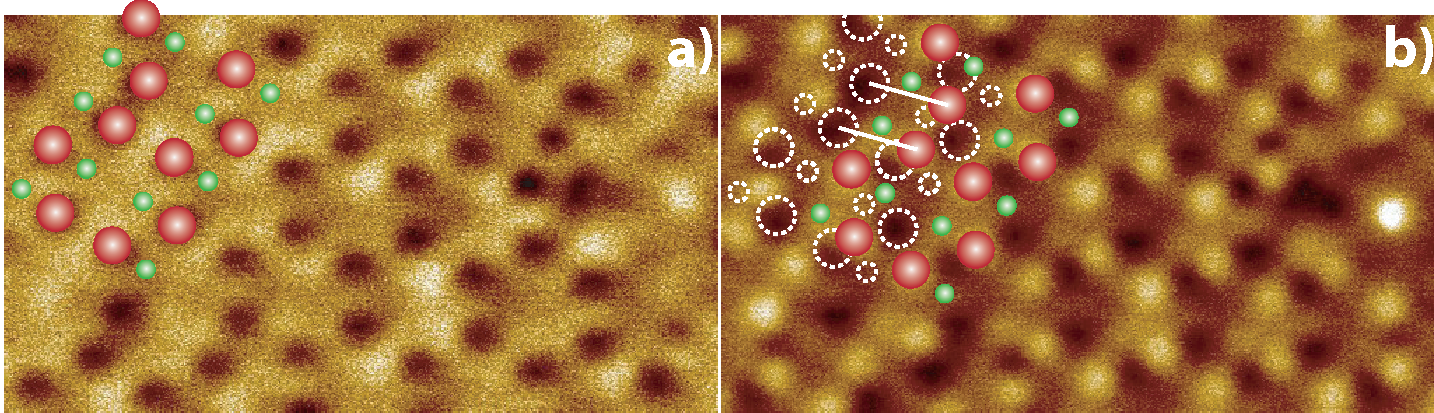

Supplement: File 3 — Atomic position assignment [file Beilstein_J_Nanotechnol-03-25-s003.png]
